# Egyptian mothers’ preferences regarding how physicians break bad news about their child’s disability: A structured verbal questionnaire

**DOI:** 10.1186/1472-6939-13-14

**Published:** 2012-07-02

**Authors:** Ahmed Mahmoud Abdelmoktader, Khalil A Abd Elhamed

**Affiliations:** 1 Department of Pediatrics, Fayoum Faculty of Medicine, Fayoum University, Al Fayoum, Egypt; 2 Faculty of Social Work, Fayoum University, Al Fayoum, Egypt

**Keywords:** Bad news, Egyptian mothers, Down syndrome

## Abstract

**Background:**

Breaking bad news to mothers whose children has disability is an important role of physicians. There has been considerable speculation about the inevitability of parental dissatisfaction with how they are informed of their child’s disability. Egyptian mothers’ preferences for how to be told the bad news about their child’s disability has not been investigated adequately. The objective of this study was to elicit Egyptian mothers’ preferences for how to be told the bad news about their child’s disability.

**Methods:**

Mothers of 100 infants recently diagnosed with Down syndrome were interviewed regarding their preferences for how to be told bad news. Mothers were recruited through outpatient clinics of the Pediatric Genetics Department at Fayoum University Hospital (located 90 km southwest of Cairo, Egypt) from January to June 2011.

**Results and discussion:**

Questionnaire analyses revealed nine themes of parental preferences for how to be told information difficult to hear. Mothers affirmed previously reported recommendations for conveying bad medical news to parents, including being told early, being told of others with a similar condition, and being informed of the prognosis.

**Conclusions:**

Mothers affirmed communication themes previously discussed in the literature, such as being told early, and being informed of the prognosis. Although more research is needed in this important area, we hope that our findings will stimulate future search and help health care providers in different societies establish guidelines for effectively communicating bad news.

## Background

To be human is to communicate. Whether we realize it or not, all of us all of the time are sending out messages to other people, directly or indirectly, about ourselves and others. [1]. Conveying bad news is one of the most important yet challenging forms of clinical communication and a skill we should be teaching routinely [2]. Communicating bad news to parents whose child has developmental or physical disabilities can be very difficult for the informer and devastating for parents. The parents’ experience in the initial informing interview can play a major role in the family’s perception of the child and their long-term adjustment to the child’s disability [3-6]. It is imperative that the informer pay careful attention to how the information is given. Despite a lengthy history in the literature that describes the best ways to inform parents of disabilities, there is some dispute about how well these practices are followed [7-9]. Early writers in this field [3,10,11] made specific recommendations for the informing interview that have been reiterated and researched over the years. Rheingold [10] emphasized the importance of the relationship between doctor and patient, and this was repeatedly stressed in the literature of the 21st century.

One of the most important Protocols for Delivering Bad News is SPIKES—A Six-Step Protocol which can achieve four essential goals. The first is gathering information from the patient. This allows the physician to determine the patient’s knowledge and expectations and readiness to hear the bad news. The second goal is to provide intelligible information in accordance with the patient’s needs and desires. The third goal is to support the patient by employing skills to reduce the emotional impact and isolation experienced by the recipient of bad news. The final goal is to develop a strategy in the form of a treatment plan with the input and cooperation of the patient [12].

In Egypt, the practice of BBN to parents involves many factors Such as the parental and family authority that is practiced in most Arabic/Islamic countries, including Egypt, experiences of the health-care professionals [13], parental preferences about BBN from Western countries [14-18] and religious factors [13], where Egyptians believe in God “Allah” destiny. Egyptian mothers’ preferences for how to be told the bad news about their child’s disability has not been investigated adequately. The objective of this cross-sectional study was to elicit Egyptian mothers’ preferences for how to be told the bad news about their child’s disability.

## Methods

We conducted interviews at the outpatient clinic of the Pediatric Genetics Department at Fayoum University Hospital from January to June 2011. The participants were a group of Egyptian mothers of children with Down syndrome. We selected 130 mothers (of the same cultural background), 100 were enrolled with response rate 77%, and 30 (23%) were excluded from the study for reasons including refusal to participate (n = 25) and previous experience with BBN (n = 5). These mothers were interviewed about their early diagnostic experience. We recruited a convenience sample from this population, using a simple random method. We used the following inclusion criteria: mothers aged 20–40 years, no previous experience with breaking bad news [BBN], not a health care provider, and able to give verbal consent.

These interviews were performed using a well-prepared structured verbal questionnaire of 20 preferences (Table 1) based on available literature and a lengthy structured interview that included questions about early diagnostic experiences. Specific questions were “What did you like about how you were told of the diagnosis?” “What didn't you like about how you were told?” “How would you want other parents to be told?” “If there is one thing that must be changed about the process of being informed of a diagnosis, what would it be?” Mothers’ responses were transcribed from initial notes during the interviews using the contrast comparative method of qualitative data analysis. The researchers examined the responses to these questions, identified key words or phrases, and grouped phrases into themes with similar meaning.

**Table 1:** Questionnaire preferences regarding how to be given bad health news about your child

No	Preference	No	Preference
1	To be alone	11	To be informed before final diagnosis
2	To be with a relative	12	To be informed after final diagnosis
3	To be informed directly	13	To be informed in front of your child
4	To be informed gradually	14	To be informed in a sympathetic way
5	To be informed with good health news only	15	To be informed by a single doctor
6	To be informed with bad health news only	16	To be informed by your doctor
7	To be informed with good and bad health news	17	To be informed by a senior doctor not related to the child’s case
8	To be informed of a prognosis	18	Confidentiality of the diagnosis
9	To be informed of others with a similar condition	19	To be informed as soon as possible in the case of newborns
10	To be informed of an approach for the diagnosis	20	To be informed by a relative

Case interviews were conducted by the researchers at the outpatient clinic of the Pediatric Genetics Department at Fayoum University Hospital, each interview lasting about 30 minutes. Mothers were interviewed alone because fathers usually did not attend or were not available (working abroad, divorced, or deceased). Mothers were informed about the objectives of the study, and the study protocol was approved by the institutional ethics committee (Fayoum Faculty of Medicine ethics committee

## Results

One hundred mothers were enrolled. Based on the interviews, twenty preferences for how to be given bad health news about their child were identified through themes related to mothers’ concerns. These themes included the following: communication of information, characteristics of the informing professional, communication of affect, pacing of the process, when told, where told, support persons present, contact with the baby, and separation of process from content. Within each of these main themes of concern, more specific and detailed subcategories were identified representing both positive and negative aspects of the interview.). Fifty children were boys and fifty were girls, 33 were the second child, 32 were the third child, 28 were first born, and seven were the fourth child. Seventy mothers were from rural areas, and 30 were from urban areas. Mothers’ ages ranged from 20 to 40 years. Educational level ranged from illiterate to high school graduate (62 went to high school, 23 graduated, 12 could read and write, and 3 were illiterate). Mothers who had graduated were younger than non-graduates. Regarding marital status, 90 were married, 7 were divorced and 3 were widows. The number of children for each mother ranged from 1 to 5 (33 mothers had 3 children, 28 had 2 children, 21 had 1 child, 12 had 4 children, and 6 had 5 children). Mothers' preferences for how to be given bad health news about their child were remarkably consistent and are summarized in Table 2.

**Table 2:** Ranking of mothers’ preferences regarding how to be given bad health news about their child

No	Preferences	%	No	Preferences	%
1	To be informed after final diagnosis	100	11	To be alone	78
2	To be informed by your doctor	100	12	To be informed with good health news only	56
3	To be informed of others with a similar condition	99	13	To be with a relative	42
4	To be informed of a prognosis	98	14	To be informed with bad health news only	42
5	To be informed in a sympathetic way	98	15	To be informed gradually	38
6	To be informed of an approach for the diagnosis	96	16	To be informed by a single doctor	26
7	To be informed directly	96	17	To be informed in front of your child	15
8	To be informed with good and bad health news	95	18	To be informed before final diagnosis	11
9	To be informed as soon as possible	94	19	To be informed by a senior doctor not related to the child’s case	10
10	Confidentiality of diagnosis	92	20	To be informed by a relative	7

## Discussion

This study is the first to examine Egyptian mothers’ preferences regarding how physicians break bad news about their child’s disability. Similar to previously reported recommendations, mothers prefer to be with fathers when told. In her early writings, Rheingold [10] recommended that parents be told together because one cannot assume that both parents have the same conception of the problem, attitudes, or level of maturity to deal with the problem. It has been reported that BBN is less stressful when it is conducted with both parents together [16]. Yet the practice has been slow to change. In 1970, Carr [19] reported that for British families, only 33% were told with both parents present. Studies of American families in the 1970s reported that 20% to 24% of parents were told together [20,21], although 86% would have preferred to be together when told [20]. In our study, mothers were interviewed alone because fathers often were not available.

Results revealed that 100% of mothers agreed that “To be informed after final diagnosis” was important, possibly because the initial response to bad news is frequently shock or disbelief. In a study of patients with cancer, the most frequent responses were shock (54%), fright (46%), acceptance (40%), sadness (24%,) and “not worried” (15%) [22]. “To be informed by your doctor” was considered important by 100% of mothers. This may reflect the trust between mother and family doctor and is consistent with the general recommendations about BBN provided by the Saudi Commission for Health Specialists (SCHS) [23]. The preference “To be informed of others with a similar condition” was deemed important by 99% of mothers, possibly indicating a mother’s desire not to be the only one who has a child with this problem. Parent-to-parent contacts often serve as a network for transmitting information and emotional support [24]. The preference “To be informed of the prognosis” was reported by 98% of mothers as important. This may reflect the mothers’ desire to know the progress of their child’s condition because hope is a strong motivator. When bad news is delivered, providing the patient with some perspective on the diagnosis is the key of providing hope so that a patient knows what the next step will be after bad news is given [25]. As noted earlier, [19,20] timing of the telling is important to families, with the large majority preferring to be told early.

Our findings show that 94% preferred to be informed as soon as possible. Studies report that parents prefer that BBN be conducted early [26]. Egyptian mothers showed preferences similar to The Saudi mothers’ and to those of their counterparts in Western countries; they preferred that BBN be conducted early, in detail, in person, and in a quiet setting [13-18]. Present results are consistent with Gayton and Walker [20] who reported that 90% of parents prefer to be told within the first week.

“To be informed in a sympathetic way” was important for 98% of mothers in this study. Although considerable attention has been given to the content of the informing interview, few researchers have looked at the affective manner in which the information is given. One early exception was Zwerling [3], who noted that some parents react with great sensitivity to the “warmth or coldness, gentleness or harshness, and the acceptance or rejection in the attitude of the doctor”. In that sample of parents, the affective tone of the communication was the second most frequently described characteristic of the informing interview. These results suggest that parents are much more sensitive to the affective attitude of the informing professional than was earlier recognized. Correspondingly, the professional's being attuned to the parent's affect may be reflected in the pacing of the diagnostic interview, with a more sensitive style of informing allowing for information to be presented at a pace that parents can follow. Importantly, this affective manner of the informer has been causally linked to parents' long-term perceptions of their child [5,21,27] and satisfaction with the process of telling [5,27,28]. “To be informed with good health news only” was considered important by 56% of mothers in this study. This reflects that our group of parents, like earlier groups, needed to hear some positive information about the child's situation [6,20]. The preference “To be informed by a senior doctor not related to the child’s case” was important to only 10% of mothers. This suggests that trust and respect between mothers and their child’s doctor is essential. However, it would be ideal if physicians uniformly offered warm, empathic responses to all of their patients [25]. Our study showed that mothers don’t like to be informed by a relative, with bad health news only and in front of their child. If there is one thing that must be changed about the process of being informed of a diagnosis, it should “To be informed by a relative”, the least preference (7%) This is consistent with the desire for confidentiality (92% of mothers preferred that), and is in agreement with recommendations for conveying bad medical news to parents [17,29-31]. It is common in our culture that relatives come with parents to hospital for support so some doctors inform relatives before parents. Also in our culture, most people considered the diseases as expiation for sins and should be confidential even for relative who may attend the process of information.

In addition to the inherent limitations of this type of study, we used a sample of convenience from one hospital, a technique which may have limited the generalizability of our findings. To help overcome this limitation, we selected our participants via a simple number-randomization scheme to reduce the selection bias from the sample. Fortunately, the demographics of our sample were similar to the overall population of Egyptian mothers [32,33]. We limited the number of questionnaire items because we did not want the participants to be overwhelmed by a lengthy questionnaire that might have decreased their overall compliance with the study. The study is action research and will mainly apply to Egyptian mothers – Though some portion of this may be applicable globally.

## Conclusions

Mothers affirmed communication themes previously discussed in the literature, such as being told early, being told of others with a similar condition, and being informed of the prognosis. Although more research is needed in this important area, we hope that our findings will stimulate future search and help health care providers in different societies establish guidelines for effectively communicating bad news.

## Competing interests

The author declares no competing interests.

## Authors’ contributions

Ahmed M. Abdelmoktader conceived of the study, participated in its design, data collection and analysis and drafted the manuscriptas as well as in the critical review of the manuscript. khalil A. Abd Elhamed conceived of the study, participated in its design, data analysis. All authors read and approved the final manuscript.

## Pre-publication history

The pre-publication history for this paper can be accessed here:

http://www.biomedcentral.com/1472-6939/13/14/prepub

## References

[B1] Bernard M Breaking Bad News 2008 Communication skills for health and social care first edition, UK:SAGE Publications Ltd 46 54

[B2] Morgan ER Winter RJ Teaching communication skills: an essential part of residency training Arch Pediatr Adolesc Med 1996 150 638 642 10.1001/archpedi.1996.02170310072013 8646316

[B3] Zwerling L psychologic aspects of pediatrics initial counseling of parents with mentally retarded children J Pediatr 1954 44 469 479 10.1016/S0022-3476(54)80222-8 13152658

[B4] Blacher J Sequential stages of parental adjustment to the birth of a child with handicaps: fact or artifact? Ment Retard 1984 22 55 68 6234448

[B5] Quine L Pahl J First diagnosis of severe mental handicap: characteristics unsatisfactory encounter between doctors and parents Soc Sci Med 1986 22 53 62 10.1016/0277-9536(86)90308-4 2937153

[B6] Lynch EC Staloch NH Parental perceptions of physicians communication in the informing process Ment Retard 1988 26 77 81 3374314

[B7] Jacobs J Improving communications between health service professionals and parents of handicapped children. A case study Br J Ment Subnorm 1977 23 54 60

[B8] Harada AE Pye PJ Handicapped babies how midwives cope Child Care Health Dev 1981 7 211 216 10.1111/j.1365-2214.1981.tb00839.x 6456849

[B9] MacDonald AC Carson KL Palmer DJ Slay T Physician's diagnostic information to parents of handicapped neonates Ment Retard 1982 20 12 14 7087785

[B10] Rheingold HL Interpreting mental retardation to parents J Consult Psychol 1945 9 162 167

[B11] Drayer C Schlesinger EG The informing interview Am J Ment Defic 1960-61 65 363 371 13724291

[B12] Baile WF Buckman R Lenzi R Glober G Beale EA Kudelka AP SPIKES—A six-step protocol for delivering bad news: application to the patient with cancer Oncologist 2000 5 302 311 10.1634/theoncologist.5-4-302 10964998

[B13] Halligan P Caring for patients of Islamic denomination: Critical care nurses’ experiences in Saudi Arabia J Clin Nurs 2006 15 12 1565 1573 10.1111/j.1365-2702.2005.01525.x 17118079

[B14] Byrnes AL Berk NW Cooper ME Marazita ML Parental evaluation of.informing interviews for cleft lip and/or palate Pediatrics 2003 112 2 308 313 10.1542/peds.112.2.308 12897279

[B15] Statham H Dimavicius J How do you give the bad news to parents? Birth 1992 19 2 103 104 10.1111/j.1523-536X.1992.tb00389.x 1388429

[B16] Krahn GL Hallum A Kime C Are there good ways to give ‘bad news’? Pediatrics 1993 91 3 578 582 8441562

[B17] Fox S Platt FW White MK Hulac P Talking about the unthinkable: perinatal/neonatal communication issues and procedures Clin Perinatol 2005 32 1 157 170 10.1016/j.clp.2004.11.011 15777827

[B18] Sharp MC Strauss RP Lorch SC Communicating medical bad news: parents’ experiences and preferences J Pediatr 1992 121 4 539 546 10.1016/S0022-3476(05)81141-2 1403386

[B19] Carr J Mongolim. Telling the parents Dev Med Child Neurol 1970 12 213 221 4246115 10.1111/j.1469-8749.1970.tb01892.x

[B20] Gayton WF Walker L Down syndrome informing the parents AJDC 1974 127 510 512 4274471 10.1001/archpedi.1974.02110230056008

[B21] Pueschel SM Murphy A Assessment of counseling practices at the birth of a child with Down's syndrome Am J Ment Defic 1976 81 325 330 138363

[B22] Butow PN Kazemi JN Beeney LJ Griffin AM Dunn SM Tattersall MH When the diagnosis is cancer: patient communication experiences and preferences Cancer 1996 77 12 2630 7 10.1002/(SICI)1097-0142(19960615)77:12<2630::AID-CNCR29>3.0.CO;2-S 8640715

[B23] Ethics of the Medical Profession Egypt demographic and health survey 2007 El-Zanaty, Fatma and Ann Way, Cairo, Egypt 11 12 http:// english.scfhs.org.sa/Book/EN-scfhs_2007_p1.pdf

[B24] Svarstad B Lipton HL Informing parents about mental retardation: a study of professional communication and parent acceptance Soc Sci Med 1977 11 645 661 10.1016/0037-7856(77)90048-8 607415

[B25] Jennifer A Harvey MD Michael A Cohen MD David R Brenin MD Brandi T Nicholson MD Reid B Adams MD Breaking Bad News: A Primer for Radiologists in Breast Imaging Journal of theAmerican College of Radiology 2007 4 11 10.1016/j.jacr.2006.07.017 17964502

[B26] Jan M Girvin JP The communication of neurological bad news to parents Can J Neurol Sci 2002 29 1 78 82 11858541 10.1017/s0317167100001773

[B27] Cunningham CC Morgan PA McGucken RB Down's syndrome is dissatisfaction with disclosure of diagnosis inevitable? Dev Med Child Neurol 1984 26 33 39 6230278 10.1111/j.1469-8749.1984.tb04403.x

[B28] Myers BA The informing interview: enabling parents to hear and cope with bad news AJDC 1983 137 572 577 6846291

[B29] Levetown M Communicating with children and families: from everyday interactions to skill in conveying distressing information Pediatrics 2008 121 e1441 e1460 10.1542/peds.2008-0565 18450887

[B30] Boyd JR A process for delivering bad news: supporting families when a child is diagnosed J Neurosci Nurs 2001 33 14 20 10.1097/01376517-200102000-00003 11233358

[B31] Garwick AW Patterson J Bennett FC Blum RW Breaking the news: how families first learn about their child’s chronic condition Arch Pediatr Adolesc Med 1995 149 991 997 10.1001/archpedi.1995.02170220057008 7655604

[B32] El-Zanaty F Way A Egypt demographic and health survey 2008

[B33] El-Zanaty F Ann W Egypt demographic and health survey Ministry of Health, El-Zanaty and Associates, and Macro International, Cairo, Egypt 2009

